# Malaria in adults after the start of Covid-19 pandemic: an analysis of admission trends, demographics, and outcomes in a tertiary hospital in the Gambia

**DOI:** 10.1186/s12936-023-04691-3

**Published:** 2023-09-01

**Authors:** Sheikh Omar Bittaye, Abubacarr Jagne, Lamin E. S. Jaiteh, Alfred Amambua-Ngwa, Abdul Karim Sesay, Bertha Ekeh, Behzad Nadjm, Williams Estrada Ramirez, Asmell Ramos, Basil Okeahialam, Emmanuel Effa, Ousman Nyan, Ramou Njie

**Affiliations:** 1 Internal Medicine Department, Edward Francis Small Teaching Hospital, Banjul, The Gambia; 2 School of Medicine and Allied Health Sciences, University of The Gambia, Banjul, The Gambia; 3 Medical Research Council, The London School of Hygiene and Tropical Medicine, Fajara, The Gambia

**Keywords:** Malaria, Adults, Trend, Admission, Outcome, COVID-19, Gambia

## Abstract

**Background:**

Malaria remains a major public health concern in The Gambia. The study assessed the trend of malaria admissions and outcome of adult patients admitted after the start of the COVID-19 pandemic in a tertiary hospital in The Gambia.

**Methods:**

This was a retrospective hospital-based study and data was collected from the 18th October 2020 to 28th February 2023. Demographic data, clinical features, investigations, treatment, and outcomes were recorded.

**Results:**

A total of 499 malaria cases were admitted to the hospital over the 29 months of the study period. Data from 320 (67.2% of the total cases) adult patients admitted into the internal medicine department were analysed. The median age was 22 years, range (15–90) and 189 (59.1%) cases were youth with a youth (15–24 years) to older adult (> 24 years) ratio of 1.4:1. The majority of the patients were male 199 (62.2) with a male to female ratio of 1.6:1. The total number of malaria cases admitted into the internal medicine department increased from 103 cases in 2021 to 182 cases in 2022and admission peaked in November in both years. The total number of admitted malaria cases during the peak of the malaria season also increased from 92 patients between September 2021 and December 2021 to 132 patients from September 2022 to December 2022.There was also an increase in both severe and uncomplicated malaria during the same period. The total mortality was 31 (9.7%) and the rate was similar in 2021 9 (8.7%) and 2022 15 (8.4%). Patients with impaired consciousness were more likely to die when compared to those without impaired consciousness [19 (23.6%) vs 12 (5%), p  ≤ 0.001]. Patients with acute kidney injury were also more likely to die when compared with those without acute kidney injury [10 (20.4%) vs 15 (7.7%), p = 0.009].

**Conclusion:**

The findings show an emerging and consistent trend of malaria admissions and the outcome in the youth and older adult population after the start of the COVID-19 pandemic in The Gambia. This, therefore, suggests the need for the implementation of targeted malaria prevention interventions in this population to further prevent the spread of the disease to the more vulnerable population.

## Background

Malaria continues to cause unacceptably high levels of morbidity and death. In 2019, there were an estimated 229,000 cases and 409,000 deaths globally with sub-Saharan Africa bearing the greatest brunt [1]. Most of the cases of malaria and related death, particularly in young children, can be attributed to *Plasmodium falciparum,* the main malaria parasite in sub-Saharan Africa. However, as transmission decreases, age immunity patterns alter, making older children more susceptible to developing severe malaria, even though the total burden of malaria may continue to fall [2, 3]. In The Gambia, severe malaria still affects young adults with significant mortality, which may have been exacerbated by the COVID-19 pandemic [4].

COVID-19 is caused by a novel beta-coronavirus that was first reported in December 2019 in the city of Wuhan, Hubei province, China [5–7]. The first case of COVID-19 was diagnosed in The Gambia on the 17th of March 2020 and as of the 13th of March 2023, 12,631 confirmed COVID-19 cases with 372 deaths have been recorded. The effects of COVID-19 on other diseases are becoming more and more obvious as it continues to dominate the health and political agendas in so many nations throughout the world [8]. Its containment strategies implemented are reported to have caused disruptions in the delivery of essential services and late diagnosis of endemic diseases, such as malaria and neglected tropical diseases (NTDs) [9, 10].

The highest incidence of clinical cases and mortality from malaria in The Gambia occurs between September and November each year [11]. The yearly incidence of malaria decreased by 43% across all seven regions in The Gambia prior to the COVID 19 pandemic, from 149 to 74 cases per 1000 people in 2011 and 2016, respectively [12]. The impact of malaria control efforts can be maximized by implementing tailored control measures to carefully selected areas and populations [13]. According to the recent Gambia national malaria indicator survey (GMIS 2017), 57% of the household population slept under an ITN the night before the survey (62% of children under age 5 and 69% of pregnant women age 15–49), 16% of households had their dwellings sprayed during the 12 months preceding the survey and 43% of women received three or more doses of fansidar for the prevention of malaria in pregnancy [12]. These interventions could also be affected by other public health events, such as COVID-19, which could influence the incidence and disease presentation.

This retrospective study was, therefore, conducted to evaluate the trend of malaria admissions and the outcome of adult patients admitted after the start of the COVID-19 pandemic in a tertiary hospital in The Gambia. This will help to identify the population at risk in The Gambia and, therefore, suggest targeted malaria prevention interventions to further prevent the spread of the disease in the vulnerable population (pregnant women and children).

## Methods

This was a retrospective hospital-based study conducted at the only tertiary hospital in The Gambia, Edward Francis Small Teaching Hospital (EFSTH), Banjul. Data was gathered from 18th October 2020 to 28thFebruary 2023**.** The EFSTH has 500-bed capacity. It serves as the primary referral centre for the nation and sees patients from across the country. The hospital is a hub for research and teaching for medical students, house and medical officers, and residents. It also provides specialized clinical services in most speciality areas and has an intensive care unit and an accident and emergency department.

This study assessed the trend of malaria admissions and the outcome of adult patients admitted after the start of the COVID-19 pandemic in a tertiary hospital in The Gambia. Patients were classified as having severe or uncomplicated malaria at the time of admission and this classification didn’t change. Severe malaria was defined according to the World Health Organization (WHO) criteria, namely, the presence of *P. falciparum* in the thick blood film (BF) or positive rapid diagnostic test (RDT), or both, in association with one or more major criteria of severity [2] (Table 1). Hyperparasitaemia and acidosis were not assessed due to limited laboratory resources. Youths were defined as those aged between 15 and 24 years and older adults above 24 years [14].

**Table 1:** Definition of the severe malaria criteria

Severe malaria criteria	Definition
Impaired consciousness	A Glasgow coma score < 11 in adults
Multiple convulsions	More than two episodes within 24 h
Hypoglycaemia	Blood or plasma glucose < 2.2 mmol/L (< 40 mg/dL)
Severe malarial anaemia	Haemoglobin concentration < 7 g/dL and < 20%, respectively, in adults
Renal impairment	Plasma or serum creatinine > 265 µmol/L (3 mg/dL) or blood urea > 20 mmol/L
Jaundice	Plasma or serum bilirubin > 50 µmol/L (3 mg/dL) with a parasite count > 100,000/µL
Significant bleeding	Including recurrent or prolonged bleeding from the nose, gums or venepuncture sites; haematemesis or melaena
Shock	Decompensated shock is defined as systolic blood pressure of < 80 mmHg in adults, with evidence of impaired perfusion (cool peripheries or prolonged capillary refill)

Laboratory diagnosis of malaria was by a rapid diagnostic test (RDT; paraHIT^®^ -f Ver1.0, Arkray, Gujarat, India) from capillary blood and/or thick blood film (BF) prepared from capillary blood, stained with Giemsa, examined under 100 × oil immersion microscopy. Haemoglobin concentrations were estimated using a HemoCuehaemoglobinometer (HemoCue 301, Angelhom, Sweden) or from full blood count usingHemax330. Blood glucose was measured at presentation in all patients using the bedside device Accu-Chek^®^active (Roche Diagnostics, Mannheim Germany). Kidney function and liver function were determined using the architect C 4000 machine.

All RDT and/or BF confirmed adult malaria patients admitted into the internal medicine department of EFSTH were included into the study. The internal medicine department had 4 wards (male and female wards, accident and emergency ward and intensive care unit ward). The study used a structured questionnaire to extract secondary data from the patient’s records, which included referral facility, demographic data, symptoms, signs, laboratory investigations, treatment, and clinical outcomes from 18th October 2020 to 28th February 2023, retrospectively. All patients of at least 15 years of age with laboratory-confirmed diagnosis of malaria by RDT and/or BF and admission to the Internal Medicine department of EFSTH were included in the study. Malaria infected pregnant women or patients with underlying severe chronic cardiac, renal, hepatic diseases or human immunodeficiency virus/acquired immunodeficiency syndrome or cerebrovascular disease, which may interfere with the evolution of the disease were excluded from analysis. The study excluded 23 patients (one patient with incomplete data, eleven patients with cerebrovascular accident, two each with congestive heart failure and human immunodeficiency virus/acquired immunodeficiency syndrome, one chronic kidney disease patient and three each with two with hyperglycaemic emergencies and chronic liver disease).

As recommended by the WHO, patients with severe malaria or those with the uncomplicated disease who were unable to tolerate oral medication were treated with intravenous infusions of artesunate at doses of 2.4 mg/kg every 12 h, 24 h, and once daily after that until they were able to tolerate oral anti-malarial (artemether-lumefantrine) therapy [2]. As a result of the increased number of malaria cases in 2021–2022, there was a stock-out of artesunate and thus intravenous quinine was used in small number of cases. Parenteral medication was stopped when the patients could swallow and a 3-day oral artemether-lumefantrine treatment was administered instead. Resuscitation and supportive management were given according to WHO guidelines [2] including correction of hypoglycaemia with 50% glucose, termination of convulsions with intravenous diazepam, blood transfusion for those with severe anaemia (haemoglobin < 7 g/dL) and haemodialysis for patients with acute kidney injury.

Microsoft Excel database (Microsoft Corp, Redmond, WA, USA) was used to enter the data. This was then imported to and analysed using STATA/SE 14.2 (Statacorp, TX, USA). Simple proportion was calculated for discrete variables. Chi-squared test and Fisher’s exact test were used for discrete variables. Statistical significance was defined as p < 0.05.

## Results

### Characteristics of the study population

A total of 499 malaria cases were admitted to the hospital during the study period. Thirty-eight cases (7.6%) were admitted into the obstetrics and gynaecology department, 118 (23.6%) cases were admitted by the paediatric department and 343 (68.7%) cases were admitted by the internal medicine department.

Out of the 343 adult patients admitted into the internal medicine department, 23 patients were excluded. Only 320 patients were evaluated and included in this study. The median age of these patients was 22 years, range (15–92) and most of the patients were within the youth group 189 (59.1%) (Table 2). Most of the patients were also male 199 (62.2). Two hundred and eight patients (65%) were referred from secondary and primary-level health facilities. The most common symptoms at presentation were fever and headache, which constituted 298 (93.1%) and 285 (89.1%) of patients, respectively. The most common signs at presentation were pallor (44.4%) and jaundice (40%). About half of the patients had severe malaria 169 (52.8%) at presentation (Table 2).

**Table 2:** Baseline characteristics of patients with malaria in EFSTH

Variable	n = 320 (%)
Age: Median (years) (range)	22 (15–92)
Age groups (years)	
15–24 (Youth)	189 (59.1)
> 24 (Older adult)	131 (40.9)
Sex (M:F)	199 (62.2): 121 (37.8)
Type of referral	
Self referral	112 ( 35)
Health facility	208 (65)
Kanifing general hospital	47 (22.6)
Bundung maternal and child hospital	53 (25.5)
Brikama district hospital	41 (19.7)
Fajikunda health centre	27 (12.9)
Essau district hospital	8 (3.9)
Others	32 (15.4)
Symptoms at presentation	
Fever	298 (93.1)
Headache	285(89.1)
Vomiting	251 (78.4)
Malaise	83 (25.9)
Abdominal pain	195 (60.9)
Convulsion	43 (13.4)
Signs at presentation	
Pallor	142 (44.4)
Jaundice	128 (40)
Median glasgow coma score at presentation	15 (3–15)
Malaria severity	
Severe malaria	169 (52.8)
Uncomplicated malaria	151 (47.2)
Outcome	
Dead	31 (9.7)
Alive	289 (90.3)

### Trend of malaria admission

The overall youth-to-older adult ratio was 1.4:1 and this reduced from 3:1 in 2020 to 1.7:1 in 2021 and 1.3:1 in 2022. The male-to-female ratio was also 1.6:1 and the ratio also decreased from 3:1 in 2020 to 2:1 in 2021 to 1.5:1 in 2022 (Table 2).

**Fig. 1:**
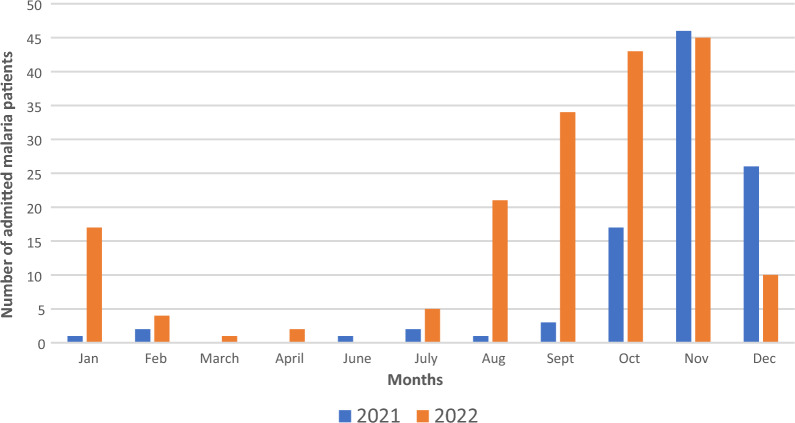
The number of patients with malaria admitted monthly in EFSTH during the years 2021 and 2022. The number of adult malaria cases admitted in the internal medicine department, EFSTH in 2021–2022. In months without a bar, no cases were admitted in that given year. October to December 2020 and January to February 2023 data were not included

**Fig. 2:**
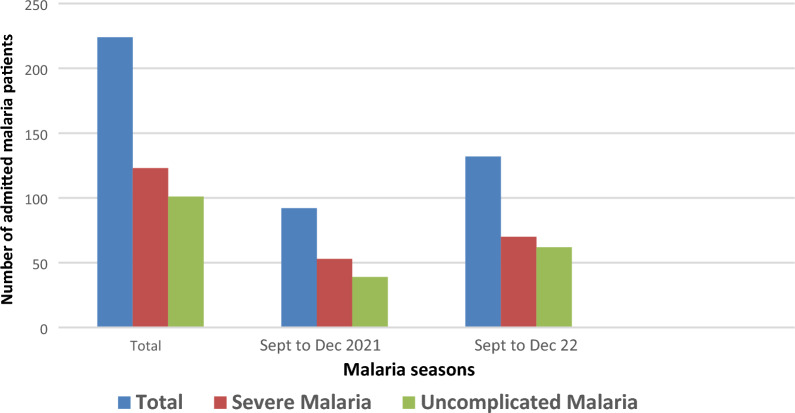
The number of patients with uncomplicated or severe malaria admitted during the peak of the malaria seasons

**Fig. 3:**
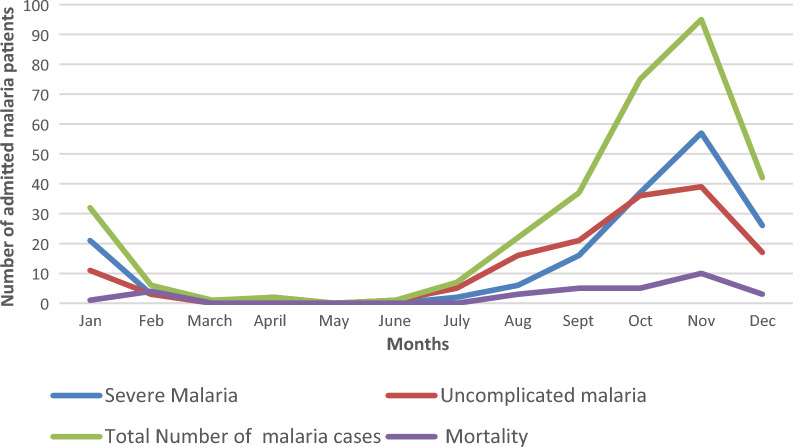
The number of patients with uncomplicated, severe malaria admitted or died monthly

The total number of malaria cases admitted in the internal medicine department in 2021 was 99 cases and 182 cases in 2022 and admission peaked in November in both years. There was also a spike in the number of admitted cases in August 2022, which corresponds to the beginning of 2022 season (Fig. 1). The total number of admitted malaria cases during the peak of the malaria season also increased from 92 patients from September 2021 to December 2021 to 132 patients for the same period in 2022. The study also showed an increase in both severe and uncomplicated malaria during this same period (Fig. 2). Severe malaria cases were predominant in the first and fourth quarters of the year and uncomplicated malaria in the third quarter of the year (Fig. 3).

### Trend of malaria outcome

The total mortality was 31 (9.7%) and 28 of them had severe malaria. Mortality in 2021 was 8.7% and this was similar to that in 20228.4%. The mortality of admitted malaria cases was high during the peak months of the malaria season (Fig. 3). Mortality was significantly higher from October 2020 to February 2021 compared to October 2021 to February 2022 and October2022 to February 2023 [6 (21.4%) vs. 7 (6.4%) vs 10 (8.9%) p = 0.05]. Higher deaths were recorded for those diagnosed with severe malaria compared to uncomplicated malaria, 28 (16.5%) vs 3 (1.9%) p < 0.001. One of the patients who died of uncomplicated malaria had parapneumonic effusion, one with hypertensive emergency and the other had a GCS of13/15 at presentation. Patients with impaired consciousness were more likely to die when compared to those without impaired consciousness 19 (23.6%) vs 12 (5%) (p ≤ 0.001). Patients with acute kidney injury (AKI) were also more likely to die when compared with those without AKI [10 (20.4%) vs 15 (7.7%) p = 0.009].

## Discussion

The study describes the trend of malaria admissions and the outcome of patients with severe malaria in EFSTH after the start of COVID-19 in The Gambia. Most of the malaria patients admitted to EFSTH during the study period were adults. The admitted adults were at least twice more as the admitted paediatric patients. This result confirms the increasing number of malaria in youths and older adults in The Gambia and is consistent with the age shift in incidence of infection towards older children which happens with endemic infectious diseases in low transmission settings [1, 15]. It is however unlike a study done in Kenya, which did not find a significant burden of severe malaria in the adults [16].

Most of the adult patients were within the youth age group but the youth-to-older adult ratio decreased overtime suggesting that more older adults also got infected after a while. This could be explained by the waning of the malarial immunity in adults following decreased exposure to parasites [15, 17–19]. There were also more male malaria patients than female and the male to female ratio also decreased over the three transmission seasons. In general, females show stronger humoral and cellular immune responses to infection or antigenic stimulation than males [20]. Gender-based occupational or behavioural factors have also been implicated in the gender differences seen in low transmission settings [15]. The COVID 19 pandemic also resulted in significant job loss across the globe and males were more affected, especially those who worked in non-agricultural industries [21, 22]. This may have resulted in lesser number of males engaging in occupations that predisposes them to malaria infections and thus contributing in the reduction of the male to female ratio over time. Other studies have also shown a high burden of malaria in adult females especially after the typical childbearing age (i.e. above 39 years of age) due to the positive health-seeking behaviours of females in these health facilities [19]. A study done during the COVID 19 pandemic also suggested changes in health behaviour with females less likely to be engaged in positive health behaviours compared to males [23]. This behavioural factor may have also resulted in the increase number of females having malaria and thus decreasing the male to female ratio over time. With less bias in the proportions in the most recent transmission season, an increasing number of older adults and females have likely either lost their immunity to malaria overtime or have not developed this due to low levels of exposure following malaria decline in The Gambia. This presents a risk to the vulnerable population (children and pregnant women), as infected adults could serve as a reservoir which spread and sustain transmission against intervention efforts. Beyond the declining transmission and incidence, other factors affecting the age shift and sex distribution of incident cases of referral malaria in The Gambia remains unclear and needs further investigation.

**Fig. 4:**
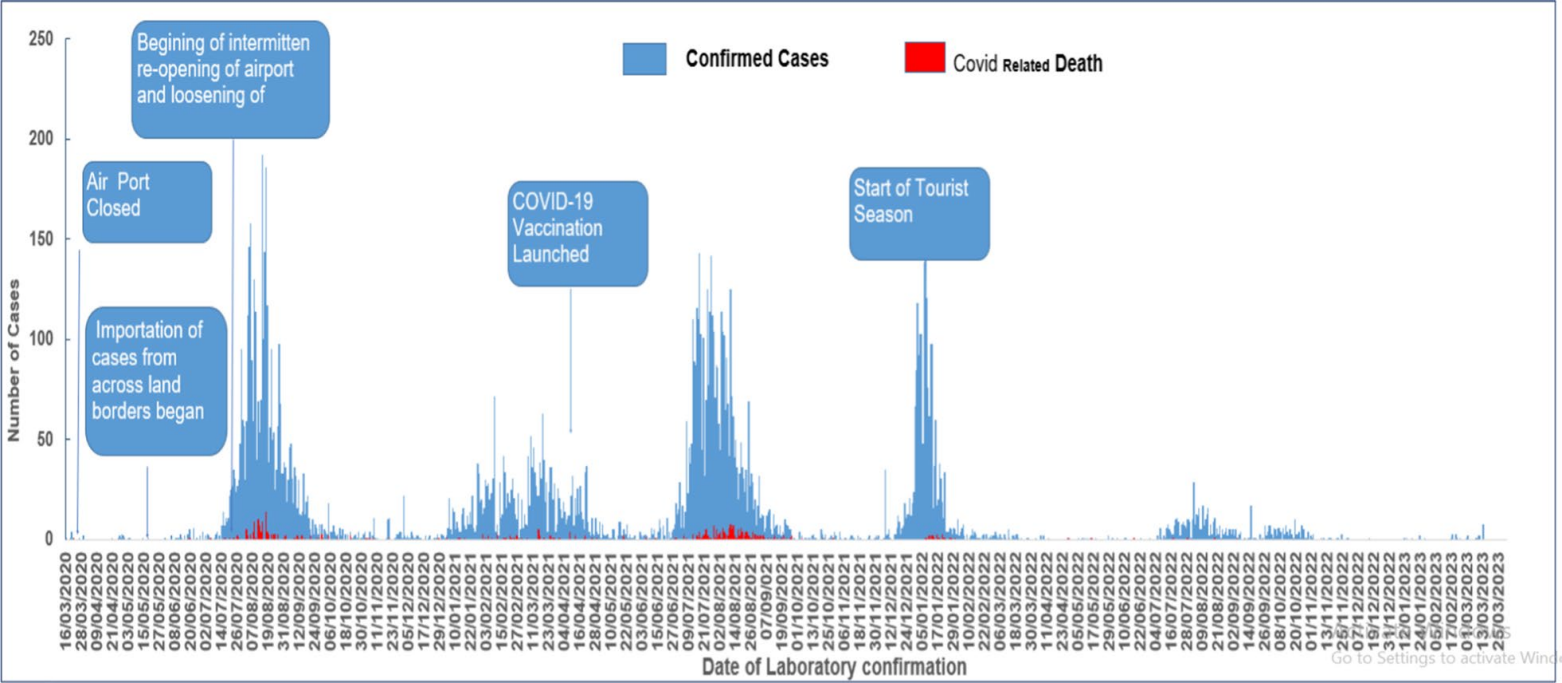
Epidemic curve of laboratory confirmed COVID 19 cases reported daily-16th March to 13th March 2023

The total number of admitted malaria cases increased by 84% from 2021 to 2022 and admission peaked in November in both years. The Gambia suffered four intensive COVID-19 waves and 3 of those were in 2021 (2nd, 3rd and beginning of the 4th waves) (Fig. 4). Out of the total number of confirmed COVID-19 cases diagnosed between 2020 and 2022, 3791 cases were diagnosed in 2020, 6681 in 2021 and 1846 cases in 2022 [24]. This showed an increase number of COVID-19 cases in 2021 as compared to 2022. This increase number of COVID-19 cases in 2021 most likely led to: (1) reduced access to healthcare services due to the pandemic control measures (such as the declaration of a state of emergency (SOE) [25], (2) a high level of fear and worry related to COVID-19 in Gambian adults [4] in the early phase of the pandemic resulting in delay in presenting to health facilities or not even visiting health facilities, (3) negative impact on the malaria preventive measures such as distribution of insecticide-treated nets (ITNs), indoor residual spraying, chemoprevention for pregnant women and young children [8, 9, 26], and (4) an undesirable effect such as the stock out of malaria test kits, reagents and medications meant for the testing and treatment of malaria [25].

There was also a spike in the number of admitted malaria cases in August 2022, which corresponds to the beginning of 2022 season. In late July and early August 2022, The Gambia suffered unprecedented torrential rainfall that resulted in widespread flooding in many communities [Greater Banjul area (Tobacco road), Ebo Town, Kotu-Manjai, and Nemakunku]. This flood was the most severe disaster the country has recorded in decades that affected more than fifty thousand people in the suburbs of urban settlements [27]. As already known, climate change also has an effect on the incidence of vector- borne diseases, such as malaria [28–30]. The increase number of admitted adult malaria patients in the early months of the 2022 malaria season most likely may have been triggered by these floods exposing this already vulnerable population to malaria infection.

The study also showed a higher number of admitted malaria cases during the peak of the malaria season in September to December 2022 as compared to September to December 2021.This jump in cases in the recent transmission season could be an indication of post- COVID-19 resurgence of malaria. It could have also been due to a decrease number of testing or diagnosis of malaria in the September to December 2021 season as majority of COVID 19 cases in The Gambia were recorded in 2021 (Fig. 4). This could have caused a disruption in testing or diagnosis of malaria resulting in decrease number of admissions in the September to December 2021 season. There is, therefore, need for increased monitoring and surveillance plus targeted preventive interventions to curb further rise in admitted malaria cases as previously suggested [4]. Malaria interventions predominantly target rural endemic areas, meanwhile, urban malaria is becoming more important. The persistent and continuous rise in malaria cases in the adult urban and peri-urban population presents a risk of spread to younger children and pregnant women, who account for most of the global mortality in higher transmission settings.

There was also an increase in both severe and uncomplicated malaria during this same period (2021 to 2022). Severe malaria cases were predominant in the first and fourth quarters of the year and uncomplicated malaria in the third quarter of the year. As patients with severe malaria are more likely to die, knowing the time the severe cases peak will help Government through the Ministry of Health and National Malaria Control Programme to deploy more preventive measures and intensify health promotion activities during this period. It could also inform decision on committing more resources (e.g. personnel, diagnostics and medications) to the health facilities during this period to prevent mortality.

The total mortality was 31 (9.7%) and mortality in 2021 and 2022 were similar. Mortality was significantly higher in October 2020 to February 2021 compared to October 2021 to February 2022 and October to February 2023. This is consistent with our previous finding as October 2020 to 2021 coincided with the end of the first wave of the COVID-19 pandemic in The Gambia [4]. Impaired consciousness using Glasgow coma score (GCS) at the time of presentation compared to those without impaired consciousness was found to be associated with death. This was also similar to the previous study [4] and has also been confirmed in several other studies in malaria-endemic and non-endemic areas [16, 31, 32]. In contrast to the previous study [4], this study also found significant relationship of death with malaria patients with AKI which is similar to the findings of other study done in African adults [31]. This adverse impact of AKI could have been due to several factors: (1) Delayed referral of patients especially those in the rural areas, (2) Limited number of critical care beds, monitoring equipment, and the capacity of personnel to provide basic critical care services such as blood transfusion at the different hospitals [33], (3) Limited access to dialysis as there is only one haemodialysis unit in the country which caters for all patients with dialysis requiring kidney failure.

One of the limitations of this study is the fact that it is a retrospective single-centre study, and the results may not be generalizable to other settings. Secondly, due to the limited access to laboratory investigations, severe malaria features such as hyperparasitaemia and acidosis could not be assessed. Notwithstanding, the study provides an understanding of temporal trends of malaria admission and outcomes in a tertiary hospital after the start of COVID-19 in The Gambia.

## Conclusion

The study shows a persistent and continuous increase of malaria in the youths and older adults after the start of the COVID-19 pandemic in The Gambia. There is, therefore, need for targeted interventions for this adult population to prevent the further spread of the disease to younger children and pregnant women which could increase the morbidity and mortality associated with malaria in The Gambia.

## Data Availability

The dataset for this publication is available on reasonable request from the corresponding author.

## Abbreviations

EFSTH: Edward Francis small teaching hospital
NTD: Neglected tropical diseases
RDT: Rapid diagnostic test
BF: Thick blood film
WHO: World Health Organization
LLN: Long‑lasting insecticidal mosquito nets
IRS: Indoor residual spraying
GCS: Glasgow coma score
COVID-19: Coronavirus 19
SOE: State of emergency
AKI: Acute kidney injury
